# Supplementary material for: CYP1-Activation and Anticancer Properties of Synthetic Methoxylated Resveratrol Analogues
Source: Molecules. 2024 Jan 15;29(2):423. doi: 10.3390/molecules29020423 (PMC10818546; doi:10.3390/molecules29020423)
Supplement: Supplementary file 1 [file molecules-29-00423-s001.zip › molecules-2775899-supplementary.pdf]

Supporting Information

Reagents were used as received from Sigma-Aldrich Chemical Company (Dorset, UK) or Alfa Aesar (Lancashire, UK).and Fisher Scientific (Loughborough, UK) The ^{1}H and ^{13}C-NMR spectra were recorded on a 400MHz super-conducting Bruker Spectrometer at 30°C. Tetramethylsilane (TMS) was used as an internal standard. Chemical shifts are reported in δ units relative to the TMS signal and coupling constants (*J*) expressed in Hertz (Hz).^{1}H-NMR information is provided in the following format: number of protons, multiplicity, coupling constant (where necessary) and assignment. Multiplicities are reported as follow; s=singlet, d=doublet, t=triplet, q=quartet, dd=doublets of doublet, m=multiplet. Infrared spectra (IR) were recorded as potassium bromide disks for solid samples and liquids as thin films using NaCl plates on a Perkin-Elmer 298 Spectrophotometer with absorption expressed in cm^{-1}. Mass spectra and Accurate mass were recorded on a Micromass Quattro II Low Resolution Triple Quadruple Mass Spectrometer (EPSRC National Mass Spectrometry Service Centre, Swansea UK). Melting points (uncorrected) were determined on a Gallenkamp melting point apparatus in open glass capillary tubes. Thin layer chromatography (TLC) was performed on Merck Aluminium Sheet- Silica Gel $60f_{254}$ coated plates. The TLC plates visualised under Multiband UVGL-58 UV-254/366nm UV light and stained with 2,4-dinitrophenylhydrazine (DNP to stain for the carbonyl group) or iodine absorbed on sand or phosphomolybdic acid (PMA). Silica gel (Fluka Silica 60; standard 30-45μ fine grade 20-45μ) was used for Flash Column Chromatography. Elemental analyses (CHN) were performed on a CE440 elemental analyser by Warwick Analytical Services and were within ±0.4% of the theoretical values, unless otherwise s noted. Carousel Reaction Station purchased (Radleys Discovery Technologies Ltd., UK) was used for in the synthesis of libraries of chalcones, pyridines and pyridines).

Stilbene synthesis

Stilbene prodrug library synthesis by Wittig reaction

General Wittig procedure for the preparation of cis- and trans-stilbenes.

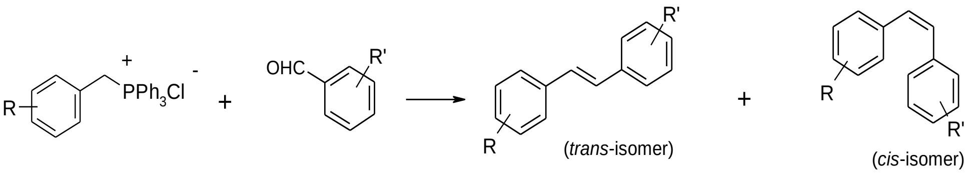

To a stirred suspension of the appropriate benzyltriphenyl phosphonium chloride (2.4mmol) in anhydrous THF (20mL), at -20°C, under nitrogen was added dropwise a solution of *n*-butyllithium in hexanes (1.49mL, 2.4mmol, 1.6M in hexane). The resulting red suspension was stirred for 20min at -20°C and then the appropriate benzaldehyde (2.4mmol), in anhydrous THF (10mL) was added dropwise. The reaction was stirred for 1h at -20°C and then allowed to warm to room temperature and stirred overnight. The reaction mixture was quenched with ice-water (40mL) and extracted with ethyl acetate (3 x 20mL). The combined organic extracts were washed with water (2 x 20mL), brine (2 x 20mL) and dried over anhydrous magnesium

sulphate. The solvent was removed *in vacuo* to afford a mixture of *cis/trans* isomers. Flash column chromatography (petroleum ether 40-60 with an increasing gradient of ethyl acetate (20-40%, silica gel 20-45micron) afforded the *cis*- and *trans*-stilbenes respectively.

(E)-4,4′-Dimethoxystilbene (DMU 205)

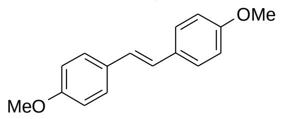

White solid (0.15g,26%), TLC: R_f 0.70 (ethyl acetate/ petroleum ether 2:8); m.p.64-67˚C; m/z [FAB] 241[M+H]$^+$,100%); υ_{MAX} (KBr)/cm^{-1} 1508, 1244, 1175, 1026, 831, 541; δ_H ($CDCl_3$) 3.75 (6H,s,2xOMe), 6.50 (2H,s,ArH), 6.80 (4H,d,*J*=17Hz,C=CH,3ArH), 7.20 (4H,d,*J*=17Hz, C=CH,3ArH); δ_C ($CDCl_3$), 55.16, 113.59, 126.00, 128.38, 130.02, 158.52; Anal. Calcd $C_{16}H_{16}O_2$: C, 79.97; H, 6.71. Found C, 79.75; H, 6.80; HRMS found [M]$^+$ 240.1144, $C_{16}H_{16}O_2$ requires [M]$^+$ 240.1145

(Z)-4,4′-Dimethoxystilbene (DMU 265)

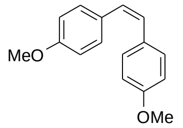

Viscous oil (0.14g,24%), TLC: R_f0.76 (ethyl acetate/ petroleum ether 2:8); m/z [FAB] 241 [M+H]$^+$, 100%); δ_H ($CDCl_3$) 3.80 (6H,s,2xOMe), 6.90 (2H, s,*J*=9Hz,C=CH,3ArH), 6.95 (4H, d, *J* =9Hz, C=CH,3ArH); δ_C ($CDCl_3$), 55.29, 114.10, 126.19, 127.38, 130.49, 159.01; Anal. Calcd $C_{16}H_{16}O_2$: C, 79.97; H, 6.71. Found C, 79.39; H, 6.81

(E)-4,3′-Dimethoxystilbene (DMU 241)

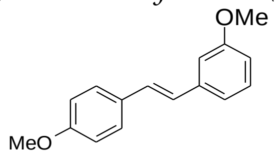

White solid (0.13g, 23%), TLC: R_f0.70 (ethyl acetate/ petroleum ether 2:8); m.p. 108-110 ˚C (lit 107-108); m/z [FAB] 241 [M+H]$^+$, 100%); δ_H ($CDCl_3$) 3.80 (3H,s,OMe), 3.82 (3H,s,OMe), 6.80 (1H,dd,ArH), 6.90 (3H,d,*J* =17Hz,C=CH, 2ArH), 7.05 (2H,m,ArH), 7.10 (1H,d,*J*=17Hz, C=CH), 7.25 (1H,t,ArH), 7.45 (2H,d,*J*=8.8Hz,C=CH,ArH).

(Z)-4,3′-Dimethoxystilbene (DMU 241C)

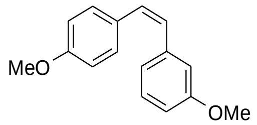

Viscous oil (0.15g, 26%), TLC: R_f0.76 (ethyl acetate/ petroleum ether 2:8); m/z [FAB] 241 [M+H]$^+$, 35%); δ_H ($CDCl_3$) 3.70 (3H,s,OMe), 3.80 (3H,s,OMe), 6.50 (2H,d,*J*=12Hz,C=CH), 6.75 (5H,m,ArH), 7.20 (3H,m,ArH).

(E)-4-Methoxy-4′-methylstilbene (DMU 216T)

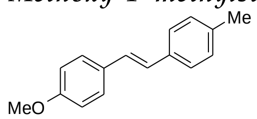

White solid, (0.21g, 39%), TLC: R_f0.17 (dichloromethane/ hexane 8:2); m.p. 84-86˚C, m/z [FAB] 225 $[M+H]^+$, 60%); δ_H ($CDCl_3$) 2.25 (3H, s, Me), 3.90 (3H, s, OMe), 6.50 (2H, s, ArH), 6.80 (2H, d, *J* =17Hz, C=CH, ArH), 7.10 (2H, d, *J* =17Hz, C=CH, ArH), 7.20(4H, m, ArH); δ_C ($CDCl_3$), 21.18, 55.15, 113.56, 113.70, 128.69, 128.74, 128.88, 129.84, 134.60,136.59, 158.59; HRMS found $[M+H]^+$ 225.1273, $C_{16}H_{16}O$ requires $[M+H]^+$ 225.1274

(Z)- 4-Methoyx- 4′-methystilbene (DMU 216C)

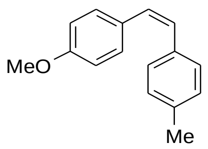

Viscous oil (0.09g, 17%), TLC: R_f0.26 (dichloromethane/ hexane 8:2); m/z [FAB] 225 $[M+H)^+$, 50%); δ_H ($CDCl_3$) 2.35 (3H, s, Me), 3.90(3H, s, OMe), 7.02 (2H, d, , *J* =12Hz, C=CH, ArH), 6.90 (2H, d), 7.20(2H, d, , *J* =12Hz, C=CH ArH), 7.40(4H, m, ArH); δ_C ($CDCl_3$), 21.16, 55.29, 114.10, 126.13, 126.57, 127.22, 129.30, 130.36, 134.86, 137.00, 159.15; HRMS found $[M+H]^+$ 225.1273, $C_{16}H_{16}O$ requires $[M+H]^+$ 225.1274; Anal. Calcd $C_{16}H_{16}O$: C, 85.68; H, 7.19. Found C, 85.62; H, 7.04

(E)-3′,4,5′-Trimethoxystilbene (DMU 271)

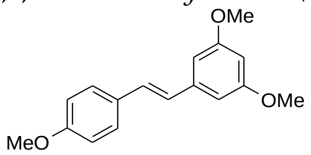

White solid (0.22g, 34%), TLC: R_f0.65 (ethyl acetate/ petroleum ether 1:9); m.p.56–57˚C (lit 57˚C[273], m/z [FAB] 271 $[M+H]^+$, 45%); δ_H ($CDCl_3$) 3.80 (9H,s,3xOMe), 6.40 (1H,t,ArH), 6.65 (2H,d,ArH), 6.85 (1H,d,*J*=16Hz,C=CH), 6.90 (2H,d,ArH), 7.10 (1H,d,*J*=16Hz,C=CH), 7.4 (2H,d,ArH); δ_C ($CDCl_3$), 55.37, 55.40, 99.67, 104.38, 113.69, 126.62, 127.86, 128.33, 128.79, 129.97, 139.75,159.45, 161.02; Anal. Calcd $C_{17}H_{18}O_3$: C, 75.53; H, 6.71. Found C, 75.60; H, 6.67; HRMS found $[M+H]^+$ 271.1329, $C_{17}H_{19}O_3$ requires $[M+H]^+$ 271.1329

(Z)-3′,4,5′-Trimethoxystilbene (DMU 210)

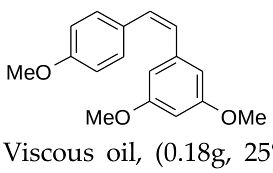

Viscous oil, (0.18g, 25%), TLC: R_f0.77 (ethyl acetate/ petroleum ether 1:9, m/z [FAB] 271 $[M+H)^+$, 56%); δ_H ($CDCl_3$) 3.60 (6H,s,2xOMe), 3.80(3H,s,OMe), 6.30 (1H,t,ArH), 6.40 (1H,d,*J*=12Hz,C=CH), 6.45 (2H,d,ArH), 6.50 (1H,d,*J*=12Hz,C=CH), 6.80 (2H,dd,ArH), 7.20 (2H,d, ArH); δ_C ($CDCl_3$), 21.16, 55.29, 114.10, 126.13, 126.57, 127.22, 129.30, 130.36, 134.86, 137.00, 159.15; Anal. Calcd $C_{17}H_{18}O_3$: C, 75.53; H, 6.71. Found C, 75.66; H, 6.78; HRMS found $[M+H]^+$ 271.1329, $C_{17}H_{19}O_3$ requires $[M+H]^+$ 271.1329

(E)-3′,4′,5′-trimethoxystilbene (DMU 507)

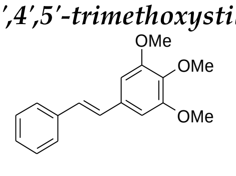

White solid, (0.15g, 23%), TLC: R_f0.70 (ethyl acetate/ petroleum ether 2:8); m.p. 108–111°C, m/z [FAB] 271 ($[M+H]^+$, 100%); δ_H ($CDCl_3$), 3.72 (3H,s,OMe), 3.75 (6H,s,2xOMe), 6.38 (2H,s,ArH), 7.00 (2H,d,CH=CH,*J*=17Hz), 7.10 (1H,t,ArH), 7.30 (2H,m,ArH),7.40 (2H,d,ArH); δ_C ($CDCl_3$), 56.30, 56.60, 105.10, 124.80, 126.20, 127.70, 128.40, 129.20, 132.40, 134.90, 148.50; HRMS found $[M+H]^+$ 271.1325, $C_{17}H_{19}O_3$ requires $[M+H]^+$ 271.1329

(Z)-3′,4′,5′-trimethoxystilbene (DMU 508)

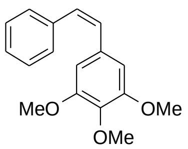

Viscous oil, (0.11g, 17%), TLC: R_f0.75 (ethyl acetate/ petroleum ether 2:8); Mass Spectrum m/z [FAB] 271 ($[M+H]^+$), 100%); δ_H ($CDCl_3$), 3.70 (3H,s,OMe), 3.72 (6H,s,2xOMe), 6.30 (2H,s,ArH), 6.50 (2H,d,CH=CH,*J*=11Hz), 6.90 (1H,t,ArH), 7.25 (2H,m,ArH),7.32 (2H,d,ArH); HRMS found $[M+H]^+$ 271.1329, $C_{17}H_{19}O_3$ requires $[M+H]^+$ 271.1329

(E)-4,3′,4′,5′-Tetramethoxystilbene (DMU 212)

Synthesis of DMU 212 precursors: 3,4,5-trimethoxybenzyltriphenylphosphonium chloride via *3,4,5-trimethoxybenzylchloride*

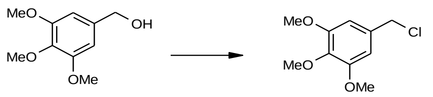

Anhydrous carbon tetrachloride (23.3g, 150 mmoles) was added to a stirred solution of 3,4,5-trimethoxybenzyl alcohol (5g, 25mmol) and triphenylphosphine (13.2g, 50 mmoles) in anhydrous dichloromethane (20mL) under nitrogen at room temperature. The mixture was stirred for 72h. The solvent was removed *in vacuo*. Flash chromatography (petroleum ether 40-60/ethyl acetate, 9:1) afforded 3,4,5-trimethoxybenzyl chloride as white solid (2.87g, 53%).

Synthesis of 3,4,5-trimethoxybenzyltriphenylphosphonium chloride

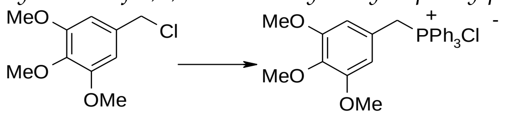

Triphenylphosphine (3.72g, 14 mmoles) was added to a solution of 3,4,5-trimethoxybenzyl chloride (2.87g, 13 mmoles) in toluene (30mL) was added The resulting solution was heated at reflux for 2h. The resulting suspension was allowed to cool to room temperature and then stirred for a further 24h. The phosphonium salt was collected by filtration, washed with diethyl ether (20mL) and dried to yield a white powder (4.65g, 73%); δ_H ($CDCl_3$), 3.40 (6H,s,2xOCH_3), 3.80 (3H,s,OCH_3), 5.5 (2H,d,$PhCH_2$), 6.5 (2H,s,ArH), 7.8 (15H,m,3x(PhH)); δ_C ($CDCl_3$), 56.09, 60.89, 108.73, 108.82, 117.47, 118.84, 122.64, 122.79, 129.94, 130.14, 134.54, 134.70, 134.79, 134.84, 153.05, 153.11

(E)-4,3′,4′,5′-Tetramethoxystilbene (DMU 212)

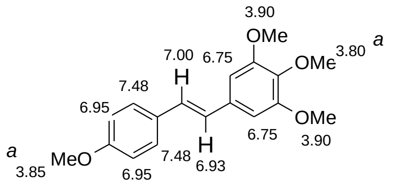

a, assignments may be interchanged

White crystals (0.20g, 28%), m.p. 158-160 °C (lit.157 °C[274]); TLC: R_f0.69 (ethyl acetate/ petroleum ether 4:6); m/z [FAB] 301 $[M+H]^+$, 100%), δ_H ($CDCl_3$) 3.80 (3H,s,OMe) 3.85 (3H,s,OMe), 3.90 (6H,s,2xOMe), 6.75 (2H,s,ArH-2′,ArH-6′), 6.93 (1H,d,*J*=16.6Hz,C=CH), 6.95 (2H,m,*J*=8.7Hz,ArH-3′,ArH-5′), 7.00 (1H,d,*J*=16.6Hz,HC=C), 7.48(2H,d,*J*=8.7Hz,ArH-2,Ar-H-6); δ_C ($CDCl_3$), 55.35 (3′,5′-OCH_3), 56.19 (4′-OCH_3), 60.97(4-OCH_3), 103.53, 114.22, 126.65, 127.67, 127.81, 130.10, 133.48, 137.86(4-C), 153.47(3′,5′-C) 159.39(4′-C); Anal. Calcd $C_{18}H_{20}O_4$: C, 71.98; H, 6.71.Found C, 71.66; H, 6.75. HRMS found $[M+H]^+$ 301.1434, $C_{18}H_{21}O_4$ requires $[M+H]^+$ 301.1434

(Z)- 4,3′,4′,5′-Tetramethoxystilbene (DMU 213)

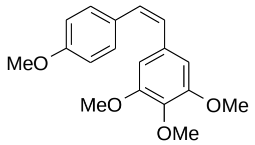

Viscous oil, (0.14g, 19%); TLC: R_f0.73 (ethyl acetate/ petroleum ether 4:6); m/z [FAB] 301 $[M+H]^+$, 100%), δ_H ($CDCl_3$) 3.70 (6H,s,2xOMe) 3.80 (3H,s,OMe), 3.90 (3H,s,OMe), 6.50(4H,m,ArH), 6.80 (2H,m,ArH), 7.20 (2H,m,ArH); δ_C ($CDCl_3$), 55.22, 55.89, 60.87, 106.02, 113.57, 128.68, 129.49, 129.69, 130.22, 132.85, 137.16, 152.90, 158.73; HRMS found $[M+H]^+$ 301.1431, $C_{18}H_{21}O_4$ requires $[M+H]^+$ 301.1434; Anal. Calcd $C_{18}H_{20}O_4$: C, 71.98; H, 6.71. Found C, 71.63; H, 6.79

(E)-4,2′,3′,4′-Tetramethoxystilbene (DMU 547)

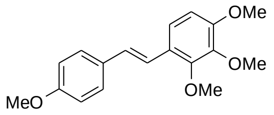

White solid (0.12g, 17%), m.p. 119-120 °C; TLC: R_f0.65 (ethyl acetate/ petroleum ether 2:8); m/z [FAB] 301 $[M+H]^+$, 15%), δ_H ($CDCl_3$) 3.80 (3H,s,OMe), 3.85 (3H,s,OMe), 3.87 (3H,s,OMe), 3.88 (3H,s,OMe), 6.70 (1H,d,*J*=16Hz,C=CH), 6.90 (2H,m,ArH), 7.00 (1H,d,*J*=16.6Hz,C=CH), 7.30 (2H,m,ArH), 7.50 (2H,d,ArH); HRMS found $[M+H]^+$ 301.1436, $C_{18}H_{21}O_4$ requires $[M+H]^+$ 301.1434

(Z)-4,2′,3′,4′-Tetramethoxystilbene (DMU 548)

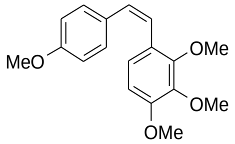

Viscous oil (0.3g, 42%);TLC: R_f0.71 (ethyl acetate/ petroleum ether 2:8); m/z [FAB] 301 $[M+H]^+$, 20%), δ_H ($CDCl_3$) 3.80 (3H,s,OMe), 3.85 (3H,s,OMe), 3.90(3H,s,2xOMe), 3.95(3H,s,OMe), 6.50 (3H,s,ArH), 6.70 (2H,d,*J*=11.4Hz,C=CH, ArH), 6.90 (1H,d,*J*=11.4Hz, C=CH), 7.20

(2H,d,ArH); δ_C ($CDCl_3$), 55.18, 55.96, 61.01, 61.08, 107.17, 113.54, 123.70, 124.35, 124.47, 129.32, 129.91, 130.10, 142.28, 152.03, 152.90, 158.54.

(E)- 4-Ethoxy-3′,4′,5′-trimethoxystilbene (DMU 260)

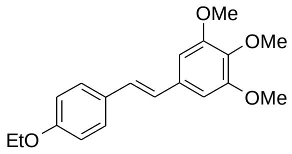

White crystals (0.24g, 32%), m.p. 87-88 °C; TLC: R_f 0.69 (ethyl acetate/ petroleum ether 4:6); m/z [FAB] 315 $[M+H]^+$; δ_H ($CDCl_3$) 1.40(3H,t,CH_3), 3.80 (3H,s,OMe), 3.90 (6H,s,2xOMe), 4.00 (2H,q,OCH_2), 6.70 (2H,s,ArH), 6.85 (1H,d,*J*=16.3Hz,C=CH), 6.87 (2H,d,ArH), 6.90 (1H,d,*J*=16.3Hz,C=CH), 7.40 (2H,d,ArH); HRMS found $[M+H]^+$ 315.1592, $C_{19}H_{23}O_4$ requires $[M+H]^+$ 315.1591

(Z)-4-Ethoxy-3′,4′,5′-trimethoxystilbene (DMU 261)

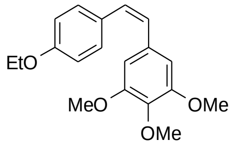

Viscous oil (0.06g, 8%); TLC: R_f 0.72 (ethyl acetate/ petroleum ether 4:6); m/z [FAB] 315 $[M+H]^+$; δ_H ($CDCl_3$) 1.40 (3H,t,CH_3), 3.70 (6H,s,2xOMe), 3.80 (3H,s,OMe), 4.00 (2H,q,OCH_2), 6.40 (4H,m,ArH), 6.80 (2H,d,*J*=12.1Hz,C=CH, ArH), 7.20 (2H,d,*J*=12.1Hz,C=CH, ArH)

(E)-4-Chloro-3′,4′,5′-trimethoxystilbene (DMU 555)

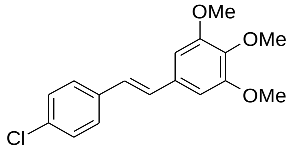

White crystals (0.13g, 18%), m.p. 146-147 °C; TLC: R_f0.40 (ethyl acetate/ petroleum ether 2:8); m/z [FAB] 305 $[M+H]^+$, δ_H ($CDCl_3$) 3.84 (6H,s,2xOMe), 3.80 (3H,s,OMe), 6.93 (1H,d,J=16.2Hz,CH=C), 6.86 (1H,d,J=16.2Hz, CH=C), 6.65 (2H,s,ArH), 7.35 (2H,d,ArH), 7.25 (2H,d,ArH); δ_C ($CDCl_3$), 56.57, 61.34, 126.16, 127.25, 127.95, 129.25, 129.70, 132.11, 133.54, 136.17, 138.75, 153.87; HRMS found $[M+H]^+$ 305.0939(Cl^{35}), $C_{17}H_{18}O_3Cl$ requires $[M+H]^+$ 305.0939(Cl^{35})

(Z)- 4-Chloro-3′,4′,5′-trimethoxystilbene (DMU 556)

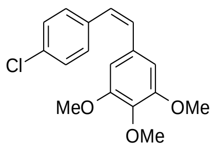

Viscous oil, (0.27g, 37%); TLC: R_f 0.50 (ethyl acetate/ petroleum ether 2:8); m/z [FAB] 305 $[M+H]^+$, δ_H ($CDCl_3$) 3.54 (6H,s,2xOMe), 3.70 (3H,s,OMe), 6.36 (1H,d,J=12.2Hz,C=CH), 6.40 (1H,d,J=12.2Hz,C=CH), 6.30 (2H,s,ArH), 7.40 (4H, m, ArH). δ_C ($CDCl_3$), 56.32, 61.29, 106.54, 128.76, 128.96, 129.25, 130.70, 131.23, 132.50, 133.21, 136.20, 153.42; HRMS found $[M+H]^+$ 305.0938 (Cl^{35}), $C_{17}H_{18}O_3Cl$ requires $[M+H]^+$ 305.0939 (Cl^{35}).

(E)- 4-Bromo-3′,4′,5′-trimethoxystilbene (DMU 557)

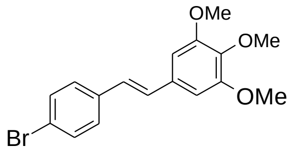

White crystals, (0.24g, 29%), TLC: R_f 0.56 (ethyl acetate/ petroleum ether 4:6); m.p. 153–154°C, m/z 348, 350 $[M]^+$ Br^{79}: Br^{81} 1:1, 80%), δ_H ($CDCl_3$) 3.55(6H, s, 2 x OMe), 3.78 (3H, s, OMe), 6.40 (1H, d, J=16Hz, C=CH), 6.48(1H, d, J=16Hz, C=CH), 6.30 (2H, s, ArH), 7.40 (4H, m, ArH). HRMS found $[M]^+$ 348.0352(Br^{79})., $C_{17}H_{17}O_3Br$ requires $[M]^+$ 348.0356(Br^{79})

(Z)- 4-Bromo-3′,4′,5′-trimethoxystilbene (DMU 558)

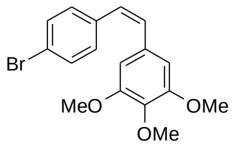

White crystals, (0.19g, 23%), TLC: R_f 0.70 (ethyl acetate/ petroleum ether 4:6); m/z 348, 350 $[M]^+$ Br^{79}: Br^{81} 1:1, 75%), %), δ_H ($CDCl_3$) 3.60(6H, s, 2 x OMe), 3.70 (3H, s, OMe), 6.35 (1H, d, J=12Hz, C=CH), 6.50(1H, d, J=12Hz, C=CH), 6.35 (2H, s, ArH), 7.20 (4H, m, ArH); HRMS found $[M]^+$ 348.0360(Br^{79}), $C_{17}H_{17}O_3Br$ requires $[M]^+$ 348.0356(Br^{79})

(E)- 4-Iodo-3′,4′,5′-trimethoxystilbene (DMU 509)

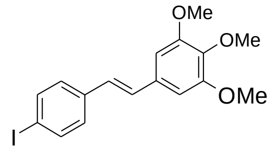

White crystals, (0.46g, 48%), m.p. 148-149 °C; TLC: R_f 0.37(ethyl acetate/ petroleum ether 2:8); m/z [EI] 396 $[M]^+$, 8%), δ_H ($CDCl_3$) 3.95(6H, s, OMe), 3.85 (3H, s, OMe), 6.70 (2H, s, ArH), 6.90(1H, d, *J*=16.24Hz, C=CH), 7.00 (1H, d, *J*=16.24Hz, C=CH), 7.70 (2H, d, ArH), 7.23 (2H, d, ArH). δ_C ($CDCl_3$), 60.95, 92.67, 103.73, 129.95, 128.10, 129.49, 132.63, 136.70, 137.34, 138.26, 153.43; HRMS found $[M]^+$ 396.0211, $C_{17}H_{17}O_3I$ requires $[M]^+$ 396.0217

(Z)- 4-Iodo-3′,4′,5′-trimethoxystilbene (DMU 510)

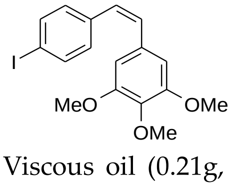

Viscous oil (0.21g, 22%); TLC: R_f 0.40 (ethyl acetate/ petroleum ether 2:8); m/z [EI] 396 ($[M]^+$, 7%), δ_H ($CDCl_3$, 400MHz) 3.85(3H, s, OMe), 3.70 (6H, s, 2 x OMe), 6.43 (2H, s, ArH), 6.46(1H, d, *J*=12, C=CH), 6.55 (1H, d, *J*=12, C=CH), 7.60 (2H, d, ArH), 7.02 (2H, d, ArH); δ_C ($CDCl_3$), 56.30, 61.30, 192.79, 106.41, 129.07, 131.22, 131.38, 132.46, 137.24, 137.70, 153.37; HRMS found $[M]^+$ 396.0214, $C_{17}H_{17}O_3I$ requires $[M]^+$ 396.0217.

(E)--1-(6-Methoxy-2-naphthyl)-2-(3,4,5-trimethoxyphenyl) ethene (DMU 567)

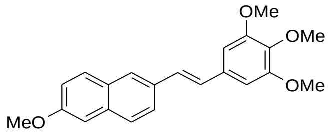

White crystals (0.14g, 17%), m.p. 142-143 °C; TLC: R_f 0.42 (ethyl acetate/ petroleum ether 2:8); m/z [EI] 350 ($[M]^+$, 100%), δ_H ($CDCl_3$) 3.90 (3H, s, OMe), 3.95 (6H, s, OMe), 4.00 (3H, s, OMe), 6.77 (2H, s, ArH), 7.09 (1H, d, *J*=16Hz, C=CH), 7.13 (2H, s, ArH), 7.16 (1H, d, J=16Hz, C=CH), 7.69 (3H, m, ArH), 7.78 (1H, s, ArH); δ_c ($CDCl_3$), 55.32, 56.13, 60.96, 103.52, 105.94, 119.05, 124.03, 126.34, 127.17, 127.93, 128.38, 129.10, 129.46, 132.61, 133.29, 134.18, 137.90, 153.42, 157.83; HRMS found $[M]^+$ 350.1520, $C_{22}H_{22}O_4$ requires $[M]^+$ 350.1513.

(Z)-1-(6-Methoxy-2-naphthyl) -2-(3,4,5-trimethoxyphenyl) ethene (DMU 568)

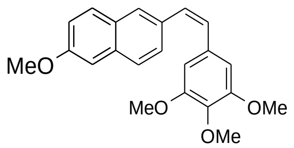

Viscous oil, (0.21g, 25%), TLC: R_f 0.56 (ethyl acetate/ petroleum ether 2:8); m/z [FAB] 351 ($[M+H]^+$, 20%); δ_H ($CDCl_3$) 4.05 (6H, s, OMe), 4.25 (3H, s, OMe), 4.35 (3H, s, OMe), 6.94 (2H, s, ArH), 6.94 (1H, d, J=12Hz, C=CH), 7.10 (1H, d, J=12Hz, C=CH), 7.50 (2H, m, ArH), 7.79 (1H, dd, ArH), 7.98 (1H, d, ArH), 8.03 (1H, d, ArH), 8.10 (1H, s, ArH); δ_C ($CDCl_3$), 55.27, 55.86, 60.89, 105.74, 106.10, 118.81, 126.25, 127.50, 127.92, 128.84, 129.35, 129.68, 129.94, 132.55, 132.70, 133.71, 137.28, 152.88, 157.81; HRMS found $[M+H]^+$ 351.1591, $C_{22}H_{23}O_4$ requires $[M+H]^+$ 351.1591

(E)-1-(4-Methoxy-1-naphthyl) -2-(3,4,5-trimethoxyphenyl) ethene (DMU 569)

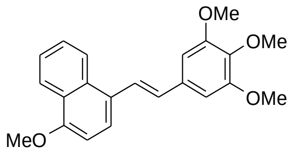

White crystals (0.18g, 21%), m.p. 146-149 °C; TLC: Rf0.66 (ethyl acetate/ petroleum ether 3:7); m/z [FAB] 351 ($[M+H]^+$, 100%), δ_H ($CDCl_3$) 3.80 (3H, s, OMe), 3.85 (6H, s, OMe), 3.95 (3H, s, OMe), 6.73 (2H, s, ArH), 6.78 (1H, d, ArH), 6.90 (1H, d, J=15Hz), 7.45 (2H, m, ArH), 7.59 (1H, m,ArH), 7.62 (1H, d, J=15Hz), 8.07 (1H, d, ArH), 8.25 (1H, d, ArH); δ_C ($CDCl_3$), 55.98, 56.62, 61.37, 72.25, 104.15, 104.30, 122.93, 123.923, 124.32, 125.60, 125.84, 126.03, 127.03, 127.89, 130.33, 132.65, 134.15, 138.43, 153.89, 155.86; HRMS found $[M+H]^+$ 351.1592, $C_{22}H_{23}O_4$ requires $[M+H]^+$ 351.1591

(Z)- 1-(4-Methoxy-1-naphthyl)-2-(3,4,5-trimethoxyphenyl) ethene (DMU 570)

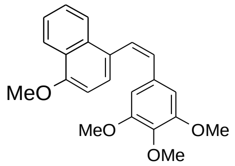

Viscous oil (0.22g, 26%); TLC: R_f 0.77 (ethyl acetate/ petroleum ether 3:7); m/z [FAB] 351 $[M+H]^+$, 10%), δ_H ($CDCl_3$) 3.35 (6H, s, OMe), 3.65 (3H, s, OMe), 4.10 (3H, s, OMe), 6.24 (2H, s, ArH), 6.59 (1H, d, J=12Hz, C=CH), 6.66 (1H, d, ArH), 6.86 (1H, d, J=12Hz, C=CH), 7.22 (1H, d, ArH), 7.40 (2H, m, ArH), 7.90 (1H, m, ArH), 8.20 (1H, d, ArH); δ_C ($CDCl_3$), 55.53, 55.58, 60.71, 103.54, 106.34, 122.23, 124.84, 125.24, 125.60, 126.33, 126.60, 127.52, 127.92, 131.28, 132.28, 137.15, 152.56, 154.94. HRMS found $[M+H]^+$ 351.1588, $C_{22}H_{23}O_4$ requires $[M+H]^+$ 351.1591

(E)- 3,4,3′,4′,5′-Pentamethoxystilbene (DMU 2417)

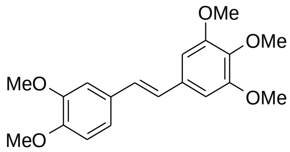

White crystals, (0.25g, 32%), m.p. 134-135 ^oC; TLC: R_f 0.69 (ethyl acetate/ petroleum ether 4:6); m/z $[EI^+]$ 330 ($[M]^+$, 100%), δ_H ($CDCl_3$) 3.80 (3H, s, OMe) 3.85 (6H, s, 2 x OMe), 3.95 (3H, s, OMe), 6.73 (2H, s, ArH), 6.90 (1H, d, J=16Hz, C=CH), 6.95 (1H, s. ArH), 6.95 (1H, d, J=16Hz, C=CH), 7.05(2H, d, ArH); δ_C ($CDCl_3$), 55.90, 56.00, 56.15, 61.03, 103.33, 108.68, 111.26, 119.85, 126.79, 128.03, 130.35, 133.34, 137.72, 148.97, 149.18, 153.44, 157.78; Anal. Calcd $C_{19}H_{22}O_5$: C, 69.08; H, 6.71. Found C, 69.06; H, 6.75; HRMS found $[M+H]^+$ 331.1538, $C_{19}H_{23}O_5$ requires $[M+H]^+$ 331.1540

(Z)-3,4,3′,4′,5′-Pentamethoxystilbene (DMU 1024)

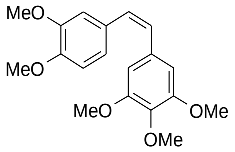

Viscous oil, (0.26g, 33%); TLC: R_f 0.73 (ethyl acetate/ petroleum ether 4:6); m/z [FAB] 331 ($[M+H]^+$, 100%), δ_H ($CDCl_3$) 3.70 (3H, s, OMe), 3.73 (6H, s, 2 x OMe), 3.80 (3H, s, OMe), 3.90(3H, s, OMe), 6.45(1H, d, J=12Hz, C=CH), 6.50 (1H, d, J=12Hz, C=CH), 6.55(2H, s, ArH), 6.60 (1H, d, ArH), 6.80(2H, m, ArH); δ_C ($CDCl_3$), 52.50, 55.53, 53.00, 57.43, 102.72, 107.08, 107.73, 116.42, 119.34, 124.41, 130.30, 138.96, 141.39, 145.80, 147.99, 149.63; Anal. Calcd $C_{19}H_{22}O_5$: C, 69.08; H, 6.71. Found C, 69.25; H, 6.73; HRMS found $[M+H]^+$ 331.1544, $C_{19}H_{23}O_5$ requires $[M+H]^+$ 331.1540

(E)-2,4,3′,4′,5′-Pentamethoxystilbene DMU 219

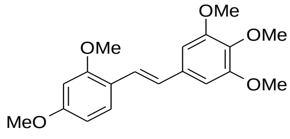

White crystals, (0.45g, 57%), m.p. 134-135 ^oC; TLC: R_f 0.69 (ethyl acetate/ petroleum ether 4:6); m/z $[EI^+]$ 330 ($[M]^+$, 100%), δ_H ($CDCl_3$) 3.70-3.80 (5 x s, 15H, 5 x OMe), 6.40 (2H, d, ArH), 6.60 (2H, s, ArH),

6.80(1H, d, J=17Hz, C=CH), 7.20 (1H, d, J=17Hz, C=CH), 7.40(1H, d, ArH); δ ($CDCl_3$) 55.39, 55.50, 56.12, 60.93, 98.52, 103.40, 104.99, 119.43, 122.87, 127.09, 127.25, 134.10, 137.50, 153.31, 157.99, 160.51.

(Z)-2,4,3′,4′,5′-Pentamethoxystilbene (DMU 222)

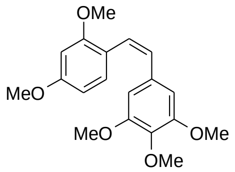

Viscous oil, (0.20g, 25%); TLC: Rf0.75 (ethyl acetate/ petroleum ether 4:6); m/z [EI$^+$] 330 ([M]$^+$, 100%), δ_H ($CDCl_3$) 3.80-4.00 (5 x s, 15H, 5 x OMe), 6.40 (1H, d, J=11Hz, C=CH), 6.50 (2H, d, ArH), 6.55 (2H, d, ArH), 6.60(1H, d, J=11Hz, C=CH), 7.30(1H, d, ArH); δ_C ($CDCl_3$), 55.37, 55.47, 55.59, 55.83, 60.65, 98.26, 104.18, 105.94, 118.77, 125.00, 128.92, 130.72, 132.99, 152.72, 158.25, 160.38.

(E)-3,4-Methylenedioxy-3′,4′,5′-trimethoxystilbene (DMU 220)

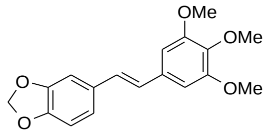

White solid (0.08g, 11%); TLC: R_f 0.60 (ethyl acetate/ petroleum ether 4:6); m.p. 176–178°C, m/z [EI$^+$] 314 ([M]$^+$, 100%), δ_H ($CDCl_3$) 3.90 (3H, s, OMe), 3.93(6H,s, 2 x OMe), 6.00 (2H, s, OCH_2O), 6.71 (2H, s, ArH), 6.82(2H, s, ArH), 6.90 (1H, d, J=17Hz, C=CH), 6.95 (1H, d, J=17Hz, C=CH), 7.08(1H, d, ArH), δ_C ($CDCl_3$), 56.10, 60.95, 101.12, 103.34, 105.44, 108.42, 121.37, 126.94, 131.73, 133.16, 148.16, 153.38, 157.45; Anal. Calcd $C_{18}H_{18}O_5$: C, 67.62; H, 5.88. Found C, 67.26; H, 5.57; HRMS found [M+H]$^+$ 315.1223, $C_{18}H_{19}O_5$ requires [M+H]$^+$ 315.1227

(Z)-3,4-Methylenedioxy-3′,4′,5′-trimethoxystilbene (DMU-299)

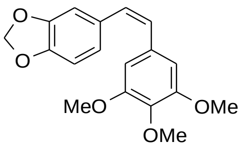

Viscous oil (0.18g, 24%); TLC: R_f 0.69 (ethyl acetate/ petroleum ether 4:6); m/z [EI$^+$] 314 ([M]$^+$, 100%), δ_H ($CDCl_3$) 3.70 (6H, s, 2 x OMe), 3.80(3H,s, OMe), 5.90 (2H, s, OCH_2O), 6.45 (1H, d, J=12Hz, C=CH), 6.50 (1H, d, J=12Hz, C=CH), 6.53(2H, s, ArH), 6.70(1H, d, ArH), 6.82(2H, d, ArH); δ_C ($CDCl_3$) δ, 55.92, 60.92, 100.91, 105.98, 108.14, 109.07, 122.91, 129.13, 131.16, 137.17, 146.59, 147.34, 152.91, 157.42; Anal. Calcd $C_{18}H_{18}O_5$: C, 68.78; H, 5.77. Found C, 69.11; H, 5.76

Stilbene synthesis by Horner-Wittig reaction

Horner-Wittig synthesis of trans- 4,3′,4′,5′-tetramethoxystilbene (DMU-212)

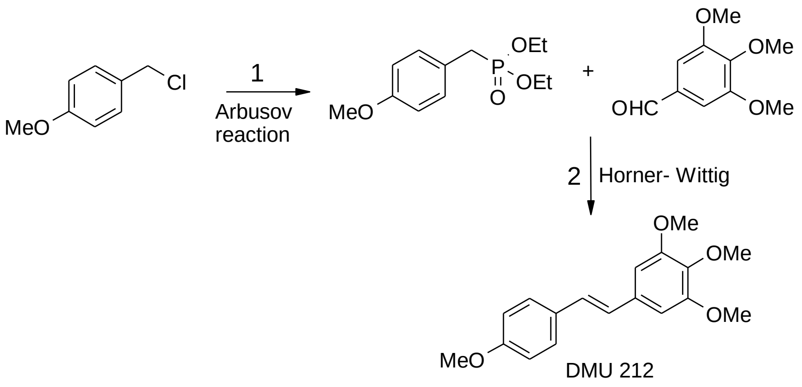

Diethyl-4-methoxybenzylphosphonate synthesis (step 1)

A mixture of 4-methoxybenzylchloride (6.39mmol) and triethyl phosphite (7.98mmol) was heated to at 110-130°C for 3h. After controlling by thin layer chromatography (TLC: Rf0.17 (ethyl acetate/ petroleum ether 2:8) that the reaction was complete, the excess triethylphosphite was removed *in vacuo* to afford resulting diethyl-4-methoxybenzylphosphonate as viscous straw coloured oil (273g, 97%).

Diethyl-4-methoxybenzylphosphonate: m/z [FAB] 259 ($[M+H]^+$, 100%);δ_H ($CDCl_3$) 7.20 (2H, d, ArH), 6.80(2H, d, ArH), 3.80-3.90(4H, m, $(OCH_2)_2$), 3.60 (3H, s, OCH_3), 3.00-2.80(2H, d, CH_2P), 1.10(6H, t, $(OCH_2C\underline{H}_3)_2$; δ_C ($CDCl_3$) 16.62, 32.34, 33.73, 55.45, 62.23, 114.29, 123.80, 130.75, 158.89; HRMS found $[M+H]^+$ 259.1096, $C_{12}H_{20}O_4P$ requires $[M+H]^+$ 259.1094.

trans- 4, 3′, 4′, 5′-Tetramethoxystilbene synthesis (step 2)

A cooled solution of diethyl-4-methoxybenzylphosphonate (5g, 19.4mmol) in DMF (50mL) was added to a stirred suspension of sodium *tert*-butoxide (38.8mmol) in DMF (50mL) at 0° C under N_2. The pale yellow solution was stirred at 0° C for a further 40min. A cooled solution of 3, 4, 5-trimethoxybenzaldehyde (3.8g, 19.4mmol) in DMF (200mL) was siphoned into the mixture. The resulting pale yellow mixture was stirred for further 1h and then allowed to warm to room temperature over 1.5h. The mixture was heated to 95 °C for 20min and then allowed to cool to room temperature. The mixture was quenched water (100mL) and the white precipitate formed was removed by filtration, washed with water (100mL) and cooled ethanol (100mL). Recrystallisation from ethyl acetate afforded DMU 212 as white crystalline solid (5.1g, 88%), m.p. 152-154 °C; TLC: R_f 0.69 (ethyl acetate/ petroleum ether 4:6); m/z [FAB] 301 ($[M+H]^+$, 100%), δ_H ($CDCl_3$) 3.8 (3H, s, OMe) 3.85 (3H, s, OMe), 3.90 (6H, s, 2 x OMe), 6.70 (2H, s, ArH), 6.85 (1H, d, J=16.59Hz), 6.90(2H, d, ArH), 6.95(1H, d, J=16.59Hz), 7.45(2H, d, ArH); δ_C ($CDCl_3$) 55.71, 56.54, 61.33, 103.88, 114.58, 126.00, 128.02, 128.17, 130.50, 133.84, 138.22, 153.83, 159.74; HRMS found $[M+H]^+$ 301.1431, $C_{18}H_{21}O_4$ requires $[M+H]^+$ 301.1434

1-(4-Methoxyphenyl)-2-(3,4,5-trimethoxyphenyl) ethane (DMU 224)

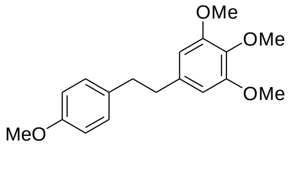

A solution of DMU 212 (0.1g, 0.3mmol) in anhydrous tetrahydrofuran (10mL) was added to a suspension of 10% palladium on carbon (0.142g, 0.13mmol) in anhydrous THF (10mL), under nitrogen. Ammonium formate (0.053g, 0.83mmol) was added and the mixture was heated at reflux for 5h. After cooling to room temperature, the catalyst was removed by filtration through celite. Removal of the solvent *in vacuo* followed by trituration with hexane yielded DMU 224 as a white solid (0.049g, 48%). TLC: R_f 0.80 (ethyl acetate/ petroleum ether 4:6), m/z ($[M]^+$, 100%), δ_H ($CDCl_3$) 7.1 (2H, dd), 6.8 (2H, dd), 6.3 (2H, s), 3.9 (9H, s), 3.8 (3H, s), 2.75 (4H, s). δ_C ($CDCl_3$), 157.9, 153.0, 137.5, 136.2, 133.7, 129.4, 113.7, 105.5, 60.8, 56.0, 55.3, 38.5, 37.0

Stilbene DMU 212 potential metabolite synthesis

(E)-3-Hydroxy-4,3′,4′,5′-tetramethoxystilbene (DMU 214)
(Z)-3-Hydroxy-4,3′,4′,5′-tetramethoxystilbene (DMU 215)

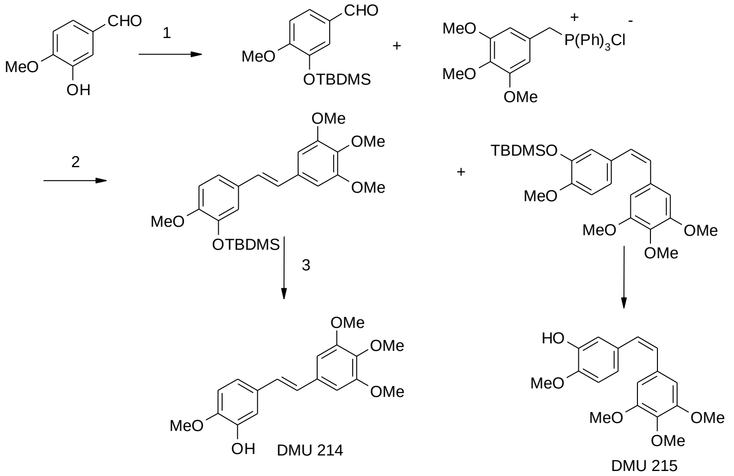

Synthesis of 3-tert-butyldimethylsiloxy-4-methoxybenzaldehyde (Step1)

tert-Butyldimethylsilyl chloride (6g, 39.6mmol) was added to a stirred solution of 3-hydroxy-4-methoxybenzaldehyde (5.0g, 33mmol) and *N,N*-diisopropylethylamine (8.5ml, 50mmol) in anhydrous DMF (100mL) under nitrogen. After 2h, the reaction was quenched with water (100mL) and extracted with ethyl acetate (3 x 100mL). The combined organic extracts were washed with saturated sodium bicarbonate (3 x 50mL), brine solution (3 x 50mL) and dried over anhydrous magnesium sulphate. The solvent was removed *in vacuo* to afford a colourless oil (8.54g 98%), m/z [FAB] 267 ($[M]^+$, 100%); δ_H ($CDCl_3$), 0.30 (6H, s, Si $(CH_3)_2$), 0.9 (9H, s, $C(CH_3)_3$), 3.80 (3H, s, OMe), 6.95 (1H, d, ArH), 7.35 (1H, d, ArH), 7.45 (H, dd, ArH), 9.00 (1H, s, CHO); δ_C ($CDCl_3$), 18.80, 26.02, 55.86, 55.95, 111.38, 111.62, 120.50, 122.74, 125.38, 126.55, 130.67, 191.22.

Synthesis of 3-(tert-butyldimethylsiloxy)-4,3′,4′,5′-tetramethoxystilbene (Step 2)

n-Butyllithium solution in 1.6M hexanes (5.51mL, 14mmol) was added dropwise to a solution of 3,4,5-trimethoxybenzyltriphenylphosphonium chloride (6g,13mmol) in anhydrous THF (100mL) at –20°C under nitrogen. The reaction was stirred for 20min after which a solution of 3-tert-butyldimethylsiloxy-4-methoxybenzaldehyde (3.33g, 10mmol) in anhydrous THF (25mL)

was added dropwise. The reaction was allowed to warm to room temperature and stirred for 3h. The reaction was quenched with ice-water (100mL) and extracted with ethyl acetate (3 x 50mL). The combined organic extracts were washed with brine solution (3 x 50mL), dried over anhydrous magnesium sulphate and the solvent removed *in vacuo*. Flash column chromatography (SiO_2, petroleum ether with an increasing gradient of ethyl acetate (0-2%) afforded a viscous oil and white solid of TBDMS-protected DMU 215 and TBDMS-protected DMU 214 respectively.

TBDMS-protected DMU 214: White solid (1.12g, 20%); δ_H ($CDCl_3$), 0.30 (6H, s, Si ($(CH_3)_2$), 0.9 (9H, s, $C(CH_3)_3$), 3.80 (3H, s, OMe), 3.85 (3H, s, OMe), 3.90 (6H, s, OMe), 6.95 (2H, s, ArH), 7.00 (3H, m, ArH), 7.20 (2H, d, ArH); δ_C ($CDCl_3$), 18.30, 25.63, 55.15, 55.46, 99.51, 106.60, 111.60, 121.35, 122.85, 128.73, 130.42, 139.63, 144.52, 150.33, 160.60.

TBDMS-protected DMU 215: Viscous oil (0.80g, 14%), δ_H ($CDCl_3$), 0.00 (6H, s, Si ($(CH_3)_2$), 0.80 (9H, s, $C(CH_3)_3$), 3.60 (6H, s, 2 x OMe), 3.70 (3H, s, OMe), 3.90 (3H, s, OMe), 6.30 (2H, d, ArH), 6.40 (2H, s, ArH), 6.60 (1H, d, ArH), 6.70 (1H, d, ArH), 6.80 (1H, d, ArH).

Synthesis of (E)-3-Hydroxy-4, 3′,4′,5′-tetramethoxystilbene (DMU 214); (Z)-3-Hydroxy-4,3′,4′,5′-tetramethoxystilbene (DMU 215) (Step 3)

A solution of tetrabutylammonium fluoride in THF (1M/THF, 2.2ml, 2.2mmol) was added to a stirred solution of *cis* or *trans* silyloxy-protected stilbene (3-(*tert*-butyldimethylsiloxy)-4,3′,4′, 5′-tetramethoxystilbene) (0.94g, 2.2mmoles) in THF (20mL) at room temperature. After 40min, the pale yellow reaction mixture was quenched with water (10mL) and extracted with ethyl acetate (3 x 5ml). The combined organic extracts were dried over anhydrous magnesium sulphate and the solvent removed *in vacuo*. Flash column chromatography (SiO_2, petroleum ether (40:60) with an increasing gradient of ethyl acetate (10-30%)) afforded DMU 214 and DMU 215 respectively. as white solid and viscous oil respectively.

(E)-3-Hydroxy-4,3′ 4′,5′-tetramethoxystilbene (DMU 214):

White solid (0.50g 73%), TLC: R_f 0.55 (ethyl acetate/ petroleum ether 4:6), m.p. 93–94˚C, m/z [FAB] 317 ($[M+H]^+$, 100%), δ_H ($CDCl_3$) 3.90 (3H, s, OMe), 3.85 (9H, s, 3 x OMe), 5.70 (1H, bs, OH), 6.7 (2H, s, ArH), 6.80 (1H, d, J= 15Hz, CH=CH), 6.90 (2H, d, J=15Hz, CH=CH), 6.95(1H, d, ArH), 7.10(1H, d, ArH). δ_C ($CDCl_3$), 53.24, 56.15, 60.49, 61.01, 103.37, 110.73, 111.80, 119.28, 127.06, 130.99, 133.40, 137.67, 145.85, 152.89; HRMS found $[M+H]^+$ 317.1386, $C_{18}H_{21}O_5$ requires $[M+H]^+$ 317.1384

(Z)-3-Hydroxy-4,3′,4′,5′-tetramethoxystilbene (DMU 215):

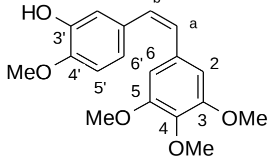

DMU 215

Viscous oil, (0.45g 75%), TLC: R_f 0.50 (ethyl acetate/ petroleum ether 4:6); m/z [FAB] 317 ($[M+H]^+$, 100%), δ_H ($CDCl_3$) 3.70 (6H, s, 3-OMe, 5-OMe), 3.80 (3H, s, 4′-OMe), 3.85 (3H, s, 4-OMe), 5.20 (1H, bs, OH), 6.40 (2H, d, J=12Hz, CH=CH), 6.50 (2H, s, ArH(2-H,6-H)), 6.70(1H, d, J=8Hz,

5′-ArH, 6.80(1H, d, J=2Hz, 6′-ArH), 6.90(1H, d, J=2Hz, 2′-ArH). δ_C ($CDCl_3$), 55.97(OMe), 55.99(OMe), 60.96(4-OMe), 106.10(C2,6), 110.35(5′-OMe), 115.07(2′-OMe), 121.15(6′-OMe), 129.08(H_a), 129.52(H_a), 132.68(C_1), 145.26, 145.78, 152.90; HRMS found $[M+H]^+$ 317.1382, $C_{18}H_{21}O_5$ requires $[M+H]^+$ 317.1384

Synthesis of (E)-4-Hydroxy-3′,4′,5′-trimethoxystilbene (DMU 281)

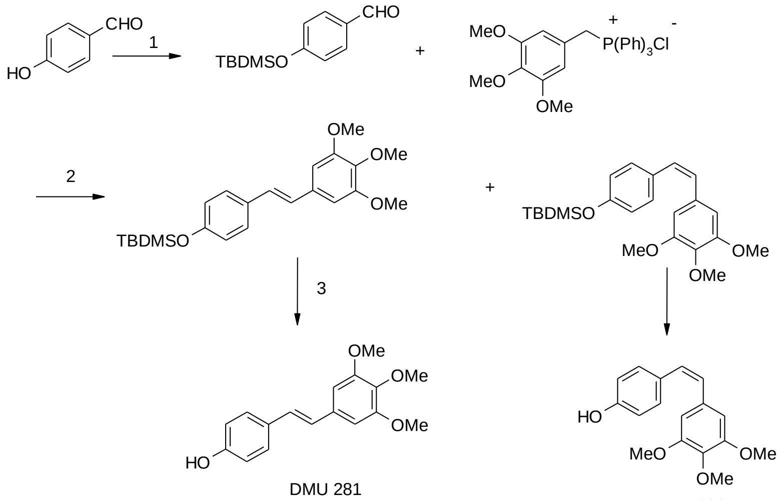

DMU 281

DMU 282

Synthesis of 4-tert-butyldimethylsiloxybenzaldehyde (Step1)

This reaction conditions are identical to that described for step1 above for the synthesis of DMU 214 using 4-hydroxybenzaldehyde(5g, 41mmol) in place of 3-hydroxy-4-methoxybenzaldehyde and *N,N*-diisopropylethylamine(10.69mL,60mmol) and *tert*-butyldimethylsilyl chloride (6g,39.6mmol) to yield 4-*tert*-butyldimethylsiloxybenzaldehyde as a viscous oil, TLC: R_f 0.77 (DCM); 9.50g, 98%), m/z [FAB] 267 ($[M]^+$, 100%); δ_H ($CDCl_3$), 0.30 (6H, s, Si $(CH_3)_2$), 0.9 (9H, s, $C(CH_3)_3$), 6.90 (2H, d, ArH), 7.85 (2H, d, ArH), 9.80 (1H, s, CHO).

Synthesis of 4-(tert-butyldimethylsiloxy)-3′,4′,5′-trimethoxystilbene (Step 2)

The reaction conditions are identical to that described for step2 above for the synthesis of DMU 214 using *n*-Butyllithium solution (2.5M in hexane, 4.60mL, 11mmol) and 3,4,5-tri methoxybenzyltriphenylphosphonium chloride (5g,11mmol) and 4-tert-butyldimethylsiloxy benzaldehyde (2.46g, 11mol) to afford 4-(tert-butyldimethylsiloxy)-3′,4′,5′-trimethoxystilbene as TBDMS-protected DMU 282 and TBDMS-protected DMU 281 respectively.

TBDMS-protected DMU 281: white solid (0.78g, 19%); TLC: R_f 0.57 (ethyl acetate/ petroleum ether 2:8); δ_H ($CDCl_3$), 0.00 (6H, s, Si $(CH_3)_2$), 0.90 (9H, s, $C(CH_3)_3$), 3.70 (3H, s, OMe), 3.80 (6H, s, OMe), 6.50 (2H, s, ArH), 6.60 (2H, d, ArH), 6.65 (1H, d, J=17.87Hz, CH=CH), 6.75 (1H, d, J=17.87Hz, CH=CH), 7.15(2H, d, ArH)

TBDMS-protected DMU 282: viscous oil (1.10g, 20%); TLC: R_f 0.71 (ethyl acetate/ petroleum ether 2:8); δ_H ($CDCl_3$) 0.20 (6H, s, Si $(CH_3)_2$), 1.00 (9H, s, $C(CH_3)_3$), 3.70 (6H, s, 2 x OMe), 3.80 (3H, s, OMe), 6.40 (1H, d, ArH), 6.50 (3H, m, ArH), 6.75 (2H, m, ArH), 7.20 (2H, d, ArH).

Synthesis of (E)-4-Hydroxy-3′,4′,5′-tetramethoxystilbene (DMU 281) (Step 3)

The reaction conditions are identical to that described for step2 above for the synthesis of DMU 214 using tetrabutylammonium fluoride (1M/THF, 0.56mL, 0.56mmol) and TBDMS- protected DMU 281 (0.23g, 0.56mmol) to afford (E)-4-Hydroxy-3′,4′,5′-tetramethoxystilbene (DMU 281) as white solid; (0.10g, 62%), m.p. 152-154 °C; TLC: R_f 0.30 (ethyl acetate/ petroleum ether 2:8); m/z [FAB] 286 ($[M+H]^+$, 75%), δ_H ($CDCl_3$) 3.80 (3H, s, OMe), 3.90 (6H, s, 2 x OMe), 5.00(1H, bs, OH), 6.70 (2H, s, ArH), 6.82 (2H, d, ArH), 6.85 (1H, d, J= 16Hz, CH=CH), 6.95(1H, d, J=16Hz, CH=CH), 7.39(2H, d, ArH); HRMS found $[M+H]^+$ 287.1276, $C_{17}H_{19}O_4$ requires $[M+H]^+$ 287.1278; Anal. Calcd $C_{18}H_{20}O_5.0.15H_2O$: C, 70.65; H, 6.38. Found C, 70.56; H, 6.56

DMU 282:

Viscous oil, TLC: R_f 0.50 (ethyl acetate/ petroleum ether 2:8); m/z [FAB] 286 ($[M]^+$, 100%), δ_H ($CDCl_3$) 3.70 (6H, s, OMe), 3.90 (3H, s, OMe), 6.38 (1H, s, ArH), 6.50 (3H, m, ArH), 6.70 (2H, d, ArH), 7.20(2H, d, ArH); HRMS found $[M+H]^+$ 287.1281, $C_{17}H_{19}O_4$ requires $[M+H]^+$ 287.1278

Synthesis of (E)-4,4′-Hydroxy-3′,5′-dimethoxystilbene (DMU 291)

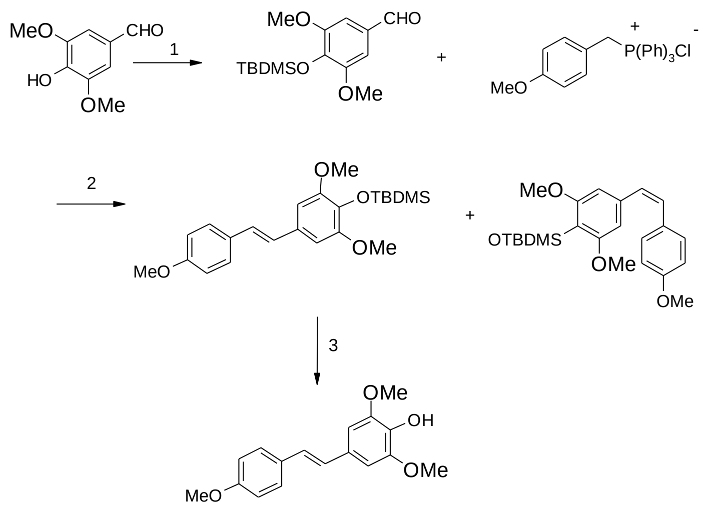

DMU 291

Synthesis of 4-tert-butyldimethylsiloxy-3,5-dimethoxybenzaldehyde (Step1)

This reaction conditions are identical to that described for step1 above for the synthesis of DMU 214 using syringaldehyde (2g,11mmol) in place of 3-hydroxy-4-methoxybenzaldehyde and *N,N*-diisopropylethylamine (2.87mL, 16mmol) and *tert*-butyldimethylsilyl chloride (1.98g,13mmol) to yield 4-*tert*-butyldimethylsiloxy-3,5-dimethoxybenzaldehyde as a viscous oil, (2.88g, 90%), m/z [FAB] 267 ($[M]^+$, 100%); δ_H ($CDCl_3$), 0.30 (6H, s, Si $(CH_3)_2$), 0.9 (9H, s, $C(CH_3)_3$), 6.90 (2H, d, ArH), 7.85 (2H, d, ArH), 9.80 (1H, s, CHO).

Synthesis of 4′-(tert-butyldimethylsiloxy)-3′,4,5′-trimethoxystilbene (Step 2)

The reaction conditions are identical to that described for step2 above for the synthesis of DMU 214 using *n*-Butyllithium solution (2.5M in hexane, 4.29mL, 11mmol) and 4-methoxybenzyltriphenylphosphonium chloride (4.08g, 10mmol) and 4-*tert*-butyldimethylsiloxy-3,5-dimethoxybenzaldehyde (2.88g, 10mmol) to afford 4′-(*tert*-butyldimethylsiloxy)-3′,4, 5′-trimethoxystilbene as *cis*-TBDMS-protected DMU 292 and *trans*-TBDMS-protected DMU 291 respectively.

TBDMS-protected DMU 291:White solid (0.60g, 62%), δ_H ($CDCl_3$) 0.20 (6H, s, Si (CH_3)$_2$), 0.9 (9H, s, C(CH_3)$_3$), 3.80 (3H, s, OMe), 3.90 (6H, s, 2 x OMe), 6.68 (2H, s, ArH), 6.86 (1H, d, J= 17.60, CH=CH), 7.90 (2H, s, ArH), 6.95 (1H, d, J= 17.60, CH=CH), 7.40(2H, d, ArH); δ_C ($CDCl_3$), δ: 23.36, 30.43, 59.89, 60.40, 108.23, 118.75, 131.17, 131.63, 132.07, 134.99, 135.09, 138.93, 156.38, 163.72.

TBDMS-protected DMU 282: viscous oil (1.10g, 20%); TLC: R_f 0.71 (ethyl acetate/ petroleum ether 2:8); δ_H ($CDCl_3$) 0.20 (6H, s, Si (CH_3)$_2$), 1.00 (9H, s, C(CH_3)$_3$), 3.70 (6H, s, 2 x OMe), 3.80 (3H, s, OMe), 6.40 (1H, d, ArH), 6.50 (3H, m, ArH), 6.75 (2H, m, ArH), 7.20 (2H, d, ArH)

Synthesis of (E)-4′-(Hydroxy)- 3′,4,5′-trimethoxystilbene (DMU 291) (Step 3)

The reaction conditions are identical to that described for step2 above for the synthesis of DMU 214 using tetrabutylammonium fluoride (1M/THF, 1.3ml, 1.3mmol) and TBDMS- protected DMU 291 (0.5g, 1.3mmol) to afford (E)-4′-(Hydroxy)-3′,4,5′-trimethoxystilbene (DMU 291) as a white solid; 0.10g, 62%; TLC: R_f 0.30 (ethyl acetate/ petroleum ether 2:8); m.p. 114–115˚C, m/z [EI] 286 ([M]$^+$, 100%), δ_H ($CDCl_3$) 3.80 (3H, s, OMe), 3.90 (6H, s, 2 x OMe), 5.50(1H, bs, OH), 6.70 (2H, s, ArH), 6.82 (2H, d, ArH), 6.85 (1H, d, J= 14Hz, CH=CH), 6.90(1H, d, J=14Hz, CH=CH), 7.43(2H, d, ArH); HRMS found [M+H]$^+$ 287.1279, $C_{17}H_{19}O_4$ requires [M+H]$^+$ 287.1278; Anal. Calcd $C_{17}H_{18}O_4.0.15H_2O$: C, 70.65; H, 6.38. Found C, 70.68; H, 6.31

Synthesis of (E)-3,4-(dihydroxy)-3′,4,5′-trimethoxystilbene (DMU 283)

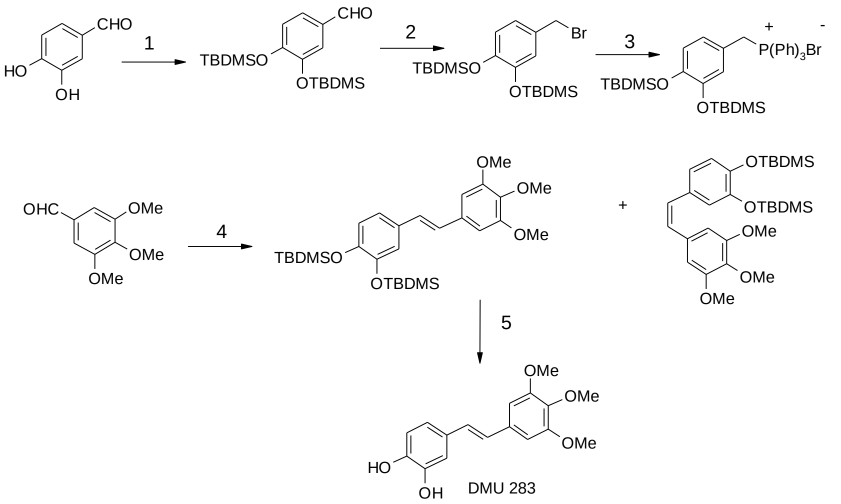

Synthesis of 3,4-bis(tert-butyldimethylsiloxy)benzaldehyde (Step1)

This reaction conditions are identical to that described for step1 above for the synthesis of DMU 214 using 3,4-dihydroxybenzaldehyde (5g, 36mmol) in place of 3-hydroxy-4-methoxy benzaldehyde and *N,N*-diisopropylethylamine (18.9ml,11mmol) and *tert*-butyldimethylsilyl chloride (13.1g,87mmol) to yield 3,4-bis(*tert*-butyldimethylsiloxy) benzaldehyde as a viscous oil; (TLC: Rf0.67 (DCM/ petroleum ether 1:1); 12.9g, 98%).

Synthesis of 3,4-bis(tert-butyldimethylsiloxy)benzylbromide (step2)

Sodium borohydride (1.46g, 38mmol) was added to 3,4-bis(*tert*-butyldimethylsiloxy) benzaldehyde (12.86g, 35mmol) in ethanol (100mL) at room temperature. The reaction was left to stir for 3h and

quenched with water (100mL) and extracted with ethyl acetate (3 x 50mL), the combined organic layers were dried over anhydrous magnesium sulphate, and the solvent removed *in vacuo*. Flash column chromatography (SiO_2, pet:ether (40:60) with an increasing gradient of ethyl acetate (0-15%)) afforded 10.03g (77%) of 3,4-bis(*tert*-butyldimethylsiloxy)benzyl alcohol as a white solid. δ_H ($CDCl_3$), 0.20 (12H, 2 x s, 2 x Si $(CH_3)_2$), 0.9 (18H, s, 2 x $C(CH_3)_3$), 6.95 (1H, d, ArH), 7.35 (2H, d, ArH), 9.80 (1H, s, CHO).

A solution of phosphorous tribromide (0.766mL, 8.2mmol) in anhydrous dichloromethane (10mL) was added dropwise to 3,4-bis(tert-butyldimethylsiloxy)benzyl alcohol (0.50g, 2.1mmol) in anhydrous dichloromethane (90mL) at 0°C under nitrogen. The reaction was stirred for 30min

and then the reaction mixture was washed sequentially with saturated sodium hydrogen carbonate (3 x 25mL), water (3 x 25mL) and brine solution (3 x 25mL). The organic extracts were dried over anhydrous magnesium sulphate and the solvent removed *in vacuo* to give 5.45g (93%) of 3,4-bis(tert-butyldimethylsiloxy)benzylbromide as oil.

Synthesis of 3,4-bis(tert-butyldimethylsiloxy)benzytriphenylphosphonium bromide (step3)

A solution of triphenylphosphine (3.6g, 13.7mmol) and 3,4-bis(tert-butyldimethylsiloxy) benzylbromide (5.38g, 12.4mmol) in toluene (100mL) was heated at reflux for 2h and the solution was allowed to cool to room temperature and stirred for 24h. A precipitate was isolated by filtration and washed with toluene and then with ether to give 3,4-bis(tert-butyldimethylsiloxy)benzytriphenylphosphoniumlbromide as a white solid (7.3g, 84.3%).

Synthesis of 3,4-bis(tert-butyldimethylsiloxy)- 3′, 4, 5′-trimethoxystilbene (step4)

The reaction conditions are identical to that described for step 2 above for the synthesis of DMU 214 using *n*-butyllithium solution (2.5M in hexane, 6.37mL, 10mmol) and 3,4-bis(*tert*-butyldimethylsiloxy)benzytriphenylphosphoniumlbromide (4.06g, 10mmol) and 3,4,5-trimethoxybenzaldehyde (2.0g, 10mmol) to afford 3,4-bis(*tert*-butyldimethylsiloxy)-3′,4,5′-trimethoxystilbene as *cis*-TBDMS-protected DMU 283 and *trans*-TBDMS-protected DMU 284 respectively. *TBDMS-protected DMU 283:* White solid (0.46g, 9%) TLC: R_f 0.41 (ethyl acetate/ petroleum ether 3:7); δ_H ($CDCl_3$), 0.05 (12H, 2 x s, 2 x Si $(CH_3)_2$), 1.00 (18H, 2 x s, 2 x $C(CH_3)_3$), 3.90 (3H, s, OMe), 3.95 (6H, s, 2 x OMe), 6.70 (2H, s, ArH), 6.80 (3H, m), 6.90 (2H, m), 7.20 (2H, d, ArH)

Synthesis of (E)-3,4-(Dihydroxy)- 3′, 4, 5′-trimethoxystilbene (DMU 283) (Step5)

The reaction conditions are identical to that described for step2 above for the synthesis of DMU 214 using tetrabutylammonium fluoride solution (1M/THF, 0.86mL, 0.86mmol) and 3,4-bis(tert-butyldimethylsiloxy)- 3′, 4, 5′-trimethoxystilbene (TBDMS- protected DMU 283; 0.46g, 0.86mmol) to afford (E)-3,4-(dihydroxy)- 3′, 4, 5′-trimethoxystilbene (DMU 283) as a white solid. *DMU 283*: White solid (0.184g, 71%); TLC: R_f 0.50 (ethyl acetate/ petroleum ether 4:6); m/z

[FAB] 303 ([M+H]$^+$, 100%); δ_H ($CDCl_3$), 3.90 (3H, s, OMe), 3.92 (6H, s, 3 x OMe), 5.35 (2H, bs, 2 x OH), 6.70 (2H, s, ArH), 6.85 (3H, m, ArH, , J=16Hz, CH=CH), 6.90(1H, d, J=16Hz, CH=CH), 7.00(1H, s, ArH), 7.10(1H, d, ArH); HRMS found [M+H]$^+$ 303.1223, $C_{17}H_{19}O_5$ requires [M+H]$^+$ 303.1227

Synthesis of (E)-4, 4′-dihydroxy- 3′, 5′-dimethoxystilbene (DMU 295) (Step1-7)

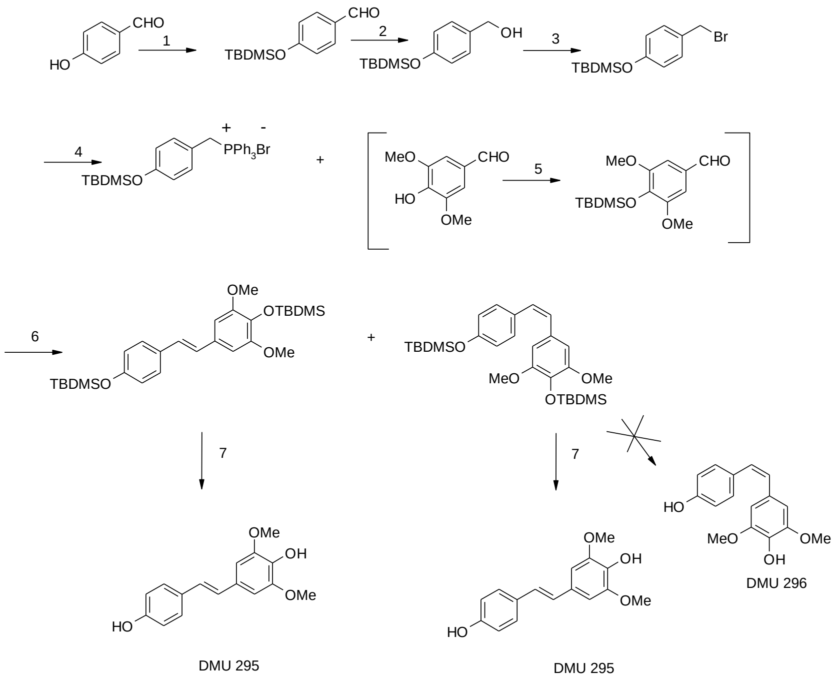

Synthesis of 4-(tert-butyldimethylsiloxy) benzaldehyde (step1)

This reaction conditions are identical to that described for step1 above for the synthesis of DMU 214 using 4-hydroxybenzaldehyde(5g, 41mmol) in place of 3-hydroxy-4-methoxybenzaldehyde and *N,N*-diisopropylethylamine (10.7ml, 60mmol) and *tert*-butyldimethylsilyl chloride (7.4g,49mmol) to yield 4-(*tert*-butyldimethylsiloxy) benzaldehyde as a viscous oil (8.6g, 45%)

Synthesis of 4-(tert-butyldimethylsiloxy) benzyl alcohol (step2)

Sodium borohydride (0.89g, 24mmol) was added to a solution of 4-(*tert*-butyldimethylsiloxy) benzaldehyde (5g, 21mmol) in ethanol. The reaction was stirred for 3h before quenching with water (100mL) and extracting with ethyl acetate (3 x 50mL). The combined organic extracts were dried over anhydrous magnesium sulphate and the solvent removed *in vacuo* to afford 4-(tert-butyldimethylsiloxy) benzyl alcohol as pale yellow oil (6.50g, 64%)

Synthesis of 4-(tert-butyldimethylsiloxy) benzyl bromide (step3)

4-(*tert*-butyldimethylsiloxy) benzyl alcohol (6g, 25mmol) in anhydrous acetonitrile (10mL) was added dropwise to a stirred solution of lithium bromide (4.38g, 50mmol) and trimethylsilylchloride (1M in dichloromethane, 63mL, 63mmol) in anhydrous acetonitrile (50mL) at under nitrogen. After 30min, the reaction mixture was washed with water (3 x 25mL) and brine solution (3 x 25mL). The organic layer was dried over anhydrous magnesium sulphate and the solvent removed *in vacuo* to give 4-(*tert*-butyldimethylsiloxy) benzyl bromide as yellow oil (6.67g, 88%).

Synthesis of 4-(tert-butyldimethylsiloxy) benzytriphenylphosphonium bromide (step4)

A solution of triphenylphosphine (6.22g, 24mmoles) and 4-(*tert*-butyldimethylsiloxy) benzyl bromide (6.67g, 22mmoles) was heated at

refluxed in toluene (50mL) for 2h and then stirred at room temperature for 24h. A precipitate was isolated by filtration and washed with toluene and then with diethyl ether to afford 4-(tert-butyldimethylsiloxy) benzytriphenylphosphonium bromide as white solid (9.37g, 75%).

Synthesis of (E)-4, 4′-bis (tert-butyldimethylsiloxy)-3′,5 ′-dimethoxystilbene (step6)

The reaction conditions are identical to that described for step2 above for the synthesis of DMU 214 using *n*-Butyllithium solution (2.5M in hexanes, 1.42mL, 3.6mmol) and 4-(*tert*-butyldimethylsiloxy)benzytriphenylphosphonium bromide (2g, 3.6mmol) and 4-(*tert*-butyldimethylsiloxy)-3,5-dimethoxybenzaldehyde (1.05g, 3.6mmol) to afford 4, 4′-bis (*tert*-butyldimethylsiloxy)—3′, 5 ′-dimethoxystilbene as *cis*-TBDMS-protected DMU 296 (4, 4′-bis (*tert*-butyldimethylsiloxy) - 3′, 5 ′-dimethoxystilbene) and *trans*-TBDMS-protected DMU 295as an oil and a white solid (0.32g, 18%) respectively. However, the *cis* isomer was discovered to be unstable and isomerised to the *trans*-isomer (DMU 295), confirmed by TLC.

Synthesis of 4, 4′-dihydroxy -3′,5′-dimethoxystilbene (DMU 295) (step7)

Tetrabutylammonium fluoride (0.10mL, 0.1mmol) was added to a solution of (E)-4, 4′-bis (*tert*-butyldimethylsiloxy) - 3′, 5 ′-dimethoxystilbene (0.05g, 0.1mmol) in THF (5ml). After 40min, the reaction was quenched with water (10mL) and extracted with ethyl acetate (3 x 5ml). The combined organic layers were dried over anhydrous magnesium sulphate and the solvent reduced *in vacuo*. Flash column chromatography (SiO_2, pet-ether (40:60) with an increasing gradient of ethyl acetate (10-30%)) afforded 4, 4′-dihydroxy) -3′,5′-dimethoxystilbene as a white solid. *DMU 295:* White solid (0.015g, 55%); TLC: R_f 0.30 (ethyl acetate/ petroleum ether 1:1); m/z [EI] 272 ($[M]^+$, 100%), δ_H ($CDCl_3$) 3.80 (6H, s, 2 x OMe), 4.70 (1H, bs, OH), 5.50 (1H, bs, OH), 6.60 (2H, s), 6.70 (2H, d), 6.80(2H, s, ArH), 7.30(2H, d); HRMS found $[M+H]^+$ 273.1120, $C_{16}H_{17}O_4$ requires $[M+H]^+$ 273.1121

Synthesis of 3, 4′-Dihydroxy-4, 3′, 5 ′-trimethoxystilbene (DMU 293) (steps 1-7)

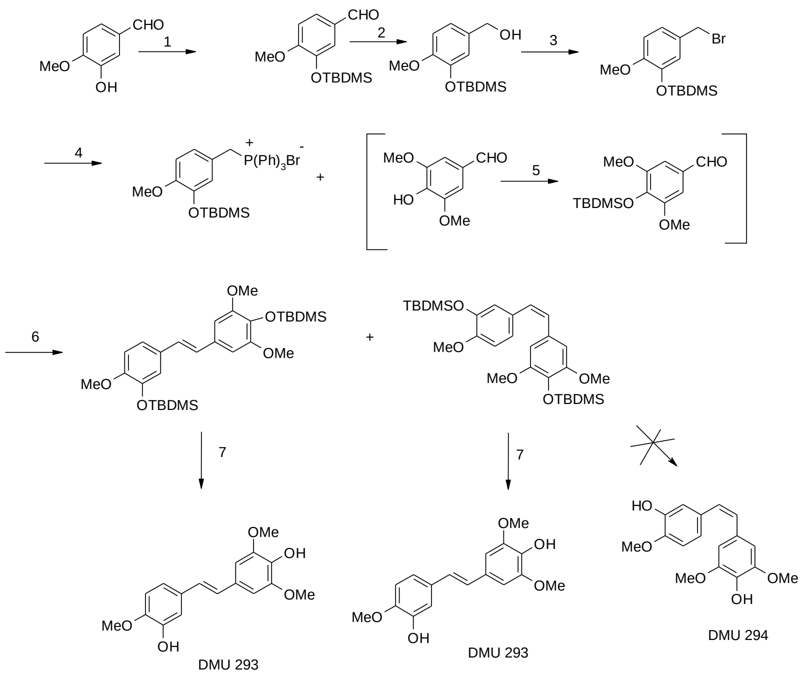

Synthesis of 3-(tert-butyldimethylsiloxy)-4-methoxybenzaldehyde (step1)

This reaction conditions are identical to that described for step1 above for the synthesis of DMU 214 using 3-hydroxy-4-methoxybenzaldehyde(5g, 33mmol) in place of 3-hydroxy-4-methoxybenzaldehyde and *N,N*-diisopropylethylamine (8.5ml, 50mmol) and *tert*-butyldimethylsilylchloride (6g,40mmol) to yield 3-(*tert*-butyldimethylsiloxy)-4-methoxy benzaldehyde as a yellow oil (8.5g, 98%).

Synthesis of 3-(tert-butyldimethylsiloxy)-4-methoxybenzyl alcohol (step2)

Sodium borohydride (0.94g, 24mmol) was added to a solution of 3-(*tert*-butyldimethylsiloxy)-4-methoxybenzaldehyde (6g, 22mmol) in ethanol. The reaction was stirred for 3h before quenching with water (100mL) and extracting with ethyl acetate (3 x 50mL). The combined organic extracts were dried over anhydrous magnesium sulphate and the solvent removed *in vacuo* to afford 3-(*tert*-butyldimethylsiloxy)-4-methoxybenzyl alcohol as pale yellow oil (5g, 84%).

Synthesis of 3-(tert-butyldimethylsiloxy)-4-methoxybenzyl bromide (step3)

3-(*tert*-butyldimethylsiloxy)-4-methoxybenzyl alcohol (4.5g, 17mmol) in anhydrous acetonitrile (10mL) was added dropwise to a stirred solution of lithium bromide (2.92g, 34mmol) and trimethylsilylchloride (1M in dichloromethane, 42mL, 42mmol) in anhydrous acetonitrile (50mL) at under nitrogen. After 30min, the reaction mixture was washed with water (3 x 25mL) and brine solution (3 x 25mL). The organic layer was dried over anhydrous magnesium sulphate and the solvent removed *in vacuo* to give 3-(*tert*-butyldimethylsiloxy)-4-methoxybenzyl bromide as yellow oil (4.05g, 73%).

Synthesis of 3-(tert-butyldimethylsiloxy)-4-methoxy benzytriphenyl phosphonium bromide (step4)

A solution of triphenylphosphine (3.17g, 12mmol) and 3-(*tert*-butyldimethylsiloxy)-4-methoxybenzyl bromide (4g, 12mmol) was heated at refluxed in toluene (50mL) for 2h and then stirred at room temperature for 24h. A precipitate was isolated by filtration and washed with toluene and then with diethyl ether to afford *3-(tert*-butyldimethylsiloxy)-4-methoxy benzytriphenylphosphonium bromide as a white solid (7g, 98%).

Synthesis of (E)-3,4′-bis (tert-butyldimethylsiloxy) 4,3′,5 ′-trimethoxystilbene (step6)

The reaction conditions are identical to that described for step2 above for the synthesis of DMU 214 using *n*-Butyllithium solution (2.5M in hexanes, 3.37mL, 8.4mmol) and *3-(tert*-butyldimethylsiloxy)-4-methoxy benzytriphenylphosphonium bromide (5g, 8.4mmol) and 4-(*tert*-butyldimethylsiloxy)-3,5-dimethoxybenzaldehyde (2.4g, 8.4mmol) to afford 3, 4′-bis (*tert*-butyldimethylsiloxy) 4, 3′, 5′-trimethoxystilbene as *cis*-TBDMS-protected DMU 294 and *trans*-TBDMS-protected DMU 293 as an oil and a white solid (0.72g, 16%) respectively. However, the *cis* isomer was discovered to be unstable and isomerised to the *trans*-isomer (DMU 293), confirmed by TLC.

Synthesis of (E)-3, 4′-Dihydroxy – 4,3′,5 ′-trimethoxystilbene (DMU 293) (step7)

Tetrabutylammonium fluoride (0.38mL, 0.37mmol) was added to a solution of (E)-3, 4′-bis (*tert*-butyldimethylsiloxy) 4, 3′, 5′-trimethoxystilbene (0.1g, 0.19mmol) in THF (5ml). After 40min, the reaction was quenched with water (10mL) and extracted with ethyl acetate (3 x 5ml). The combined organic layers were dried over anhydrous magnesium sulphate and the solvent reduced *in vacuo*. Flash column chromatography (SiO_2, pet: ether (40:60) with an increasing gradient of ethyl acetate (10-30%)) afforded (E)-3, 4′-dihydroxy – 4,3′,5′-trimethoxystilbene as a white solid (0.05g, 88%); TLC: R_f 0.55 (ethyl acetate/ petroleum ether 4:6); m/z [EI] 302 ($[M+H]^+$, 60%), δ_H ($CDCl_3$) 3.80 (3H, s, OMe), 3.90 (6H, s, 2 x OMe), 5.60 (2H, bs, 2 x OH), 6.60 (2H, s, ArH), 6.70 (3H, m), 6.80(1H, d), 7.10(1H, d); δ_C ($CDCl_3$), 53.45, 55.98, 56.29, 103.18, 110.77, 111.69, 118.91, 126.46, 128.59, 131.19, 146.29, 147.19
